# Effects of frailty, geriatric syndromes, and comorbidity on mortality and quality of life in older adults with HIV

**DOI:** 10.1186/s12877-022-03719-8

**Published:** 2023-01-03

**Authors:** Fátima Brañas, Miguel Torralba, Antonio Antela, Jorge Vergas, Margarita Ramírez, Pablo Ryan, Fernando Dronda, María José Galindo, Isabel Machuca, María Jesús Bustinduy, Alfonso Cabello, María Luisa Montes, Matilde Sánchez-Conde, FUNCFRAIL study group

**Affiliations:** 1 Geriatrics Department, Hospital Universitario Infanta Leonor, Fundación para la Investigación e Innovación Biomédica H.U Infanta Leonor y H.U. Sureste. Universidad Complutense, Madrid, Spain; 2 Internal Medicine Department, Hospital Universitario de Guadalajara. Universidad de Alcalá, Guadalajara, Spain; 3 Infectious Diseases Unit, Hospital Clínico Universitario de Santiago de Compostela, Universidad de Santiago de Compostela, Madrid, Spain; 4 Internal Medicine/ Infectious Diseases Department, Hospital Universitario Clínico San Carlos, Madrid, Spain; 5 Infectious Diseases Unit, Hospital General Universitario Gregorio Marañón, Madrid, Spain; 6 HIV Clinic. Hospital Universitario Infanta Leonor, Fundación para la Investigación e Innovación Biomédica H.U Infanta Leonor y H.U. Sureste. Universidad Complutense. CIBERINFEC, Madrid, Spain; 7 Infectious Diseases Department, Hospital Universitario Ramón y Cajal. IRYCIS. CIBERINFEC, Madrid, Spain; 8 Internal Medicine/ Infectious Diseases Department, Hospital Universitario Clínico de Valencia, Valencia, Spain; 9 Infectious Diseases Department, Hospital Universitario Reina Sofía, Córdoba, Spain; 10 Infectious Diseases Department, Hospital de Donostia, San Sebastián, Spain; 11 Infectious Diseases Department, Fundación Jiménez Díaz, Madrid, Spain; 12 HIV Unit/Internal Medicine Department, Hospital Universitario La Paz. IdiPAZ, Madrid, Spain

**Keywords:** HIV, Frailty, Geriatric syndromes, Mortality, Quality of life

## Abstract

**Background:**

To understand the effects of frailty, geriatric syndromes, and comorbidity on quality of life and mortality in older adults with HIV (OAWH).

**Methods:**

Cross-sectional study of the FUNCFRAIL multicenter cohort. The setting was outpatient HIV-Clinic. OAWH, 50 year or over were included. We recorded sociodemographic data, HIV infection-related data, comorbidity, frailty, geriatric syndromes (depression, cognitive impairment, falls and malnutrition), quality of life (QOL) and the estimated risk of all-cause 5-year mortality by VACS Index. Association of frailty with geriatric syndromes and comorbidity was evaluated using the Cochran-Mantel-Haenszel test.

**Results:**

Seven hundred ninety six patients were included. 24.7% were women, mean age was 58.2 (6.3). 14.7% were 65 or over. 517 (65%) patients had ≥3 comorbidities, ≥ 1 geriatric syndrome and/or frailty. There were significant differences in the estimated risk of mortality [(frailty 10.8%) vs. (≥ 3 comorbidities 8.2%) vs. (≥ 1 geriatric syndrome 8.2%) vs. (nothing 6.2%); *p* = 0.01] and in the prevalence of fair or poor QOL [(frailty 71.7%) vs. (≥ 3 comorbidities 52%) vs. (≥ 1 geriatric syndrome 58.4%) vs. (nothing 51%); *p* = 0.01]. Cognitive impairment was significantly associated to mortality (8.7% vs. 6.2%; *p* = 0.02) and depression to poor QOL [76.5% vs. 50%; p = 0.01].

**Conclusions:**

Frailty, geriatric syndromes, and comorbidity had negative effects on mortality and QOL, but frailty had the greatest negative effect out of the three factors. Our results should be a wake-up call to standardize the screening for frailty and geriatric syndromes in OAWH in the clinical practice.

**Trial registration:**

NCT03558438.

## Background

Older adults comprise more than half of the people living with HIV, especially in high-income countries, and this proportion is estimated to globally increase so that 23% of older persons with HIV will be 65 or older by 2030 [1, 2]. Even if the targets for reducing HIV diagnoses were met, the number of people living with HIV would fall but the proportion of older people would increase, as would the median age of people living with HIV [1]. The aging of people with HIV is already a fact, and it is critical to target clinical practice and adapt health systems to meet this population’s real needs. The term “older adults with HIV” (OAWH) encompasses a very diverse group of people with distinct characteristics and requirements, but their care is still mainly focused on addressing comorbidity in a uniform way that does not consider their varying profiles, an approach very similar to that used to treat young adults. Comorbidity related to aging has been demonstrated to be more prevalent and appear earlier among OAWH than it does in the overall population [2, 3], but important differences exist among this group according to their chronological age as well as year of HIV diagnosis [4], sex [5], and race [6]. Moreover, the World Health Organization (WHO) has defined healthy aging as a process of maintaining functional ability to enable well-being in older age [7]. According to the WHO, this process requires abandoning disease treatment as the core of medical care [8], instead considering frailty and other geriatric syndromes beyond comorbidities and focusing on quality of life (QOL). Frailty is highly prevalent among OAWH [9, 10] and is well-known to confer a high risk for all causes of mortality [11, 12]. OAWH frequently experience geriatric syndromes, too, but at a younger age than in the rest of the population [13], and geriatric syndromes have been associated with increased healthcare utilization [14], but no evidence has yet shown the possible association between geriatric syndromes and mortality.

Due to the highly active antiretroviral treatment, the average lifespan among people with virologically controlled HIV is close to that of overall population [15], but it continues to be an important clinical and research target. Because HIV infection has become a chronic disease, it is particularly important not to focus solely on survival but rather on QOL; however, the evidence in this sense is insufficient. Recently, studies have found that specific comorbidities such as hypertension [16], hepatic steatosis [17], sleep disorders [18], and depression [19, 20], negatively affect health-related QOL, but only one study has specifically assessed the effect of the burden of comorbidity on QOL [21], and only one study has examined the relationship between frailty and QOL [22]. No evidence is available about geriatric syndromes and QOL in OAWH. Frailty, geriatric syndromes, and comorbidity in OAWH have been studied separately, but their relationships and the ways they affect mortality and QOL remain unexplored. For this reason, we performed this study to understand the effects of frailty, geriatric syndromes, and comorbidity on mortality and QOL in OAWH and whether the interaction between frailty with comorbidity and/or geriatric syndromes could increase this effect.

## Methods

### Study design and patient population

We performed a cross-sectional study of the FUNCFRAIL Spanish cohort [4]. The inclusion criteria were confirmed HIV infection, age ≥ 50 years at the time of recruitment, and regular follow-up at the HIV clinic. Age 50 is the currently accepted age cutoff for defining older people with HIV [23]. We recruited 801 patients who were then included in a random way, at the HIV clinics of 11 hospitals in Spain. Complete data were available from 796 patients. The patients agreed to participate and had the ability to understand the study’s procedure and to sign a written informed consent on their own. The study was approved by the ethics committees at all the participants’ hospitals.

### Data collection

Data recorded in the FUNCFRAIL Spanish cohort [4] were sociodemographic data, HIV-infection-related data, and medications (polypharmacy defined as taking ≥ 5 comedications other than antiretroviral treatment). Frailty was defined according to Fried’s criteria [24]; namely, shrinking (unintentional weight loss of ≥ 4.5 kg or ≥ 5% of body weight during the previous year), weakness (grip strength adjusted for sex and BMI), poor endurance and low energy (self-reported exhaustion identified by two questions from the Center for Epidemiologic Studies Depression scale), slowness (based on time to walk 4.6 m, adjusting for gender and standing height), and low physical activity level (< 383 kcal/week in men and < 270 kcal/week in women using the Minnesota Leisure Time Activity Questionnaire). Patients were considered frail if they met at least three of the five criteria, prefrail when they met one or two criteria, and robust when they met no criteria.

Comorbidity was recorded based on self-reported, physician-diagnosed chronic conditions: hypertension, type 2 diabetes, dyslipidemia, coronary heart disease, stroke, COPD, chronic kidney disease, cancer (< 5 years from diagnosis), history of cancer (≥ 5 years from diagnosis; not active disease), psychiatric disorders, and osteoarticular disease.

The geriatric syndromes we recorded were falls, cognitive impairment, depression, and risk of malnutrition. To avoid overlap, we decided not to include physical impairment among the geriatric syndromes recorded because it is closely related to frailty. Falls were evaluated by self-reporting in response to the question, “Have you fallen in the past year?” Cognitive impairment was evaluated using the Montreal Cognitive Assessment (MOCA) test [25]. The cutoff point we used was < 20 points to differentiate subjects without cognitive impairment from subjects with cognitive impairment and/or dementia, including MCI [26], and multifactorial causes for cognitive impairment. Depression was tested using the Short-Form Geriatric Depression Scale [27], and the cutoff used to consider depressive symptoms was ≥ 6 points. Risk of malnutrition was evaluated according to the Mini-Nutritional Assessment Short Form [28], considering a score lower than 11 a sign of malnutrition risk.

QOL was evaluated by self-assessment: patients stratified their QOL into one of the following categories: very good, good, fair, or poor.

To estimate risk of all-cause mortality, we used the Veterans Aging Cohort Study (VACS) Index [29], which includes the following data: chronological age, CD4 lymphocytes count (cells/mm^3^), HIV-1 RNA copies/mL, hemoglobin (g/dL), estimated glomerular filtration rate (mL/min), Fibrosis-4 Index for Liver Fibrosis (age, aspartate aminotransferase U/L, alanine aminotransferase U/L, and platelet count), and hepatitis C coinfection. We used the VACS Index to predict mortality because it is considered the “gold standard” measurement for predicting mortality among OAWH [30].

### Statistical analysis

We used descriptive statistics to examine the participants’ characteristics, which are expressed as frequencies (percent) of categorical variables, mean (SD) of normally distributed continuous variables, and median (p25-p75) of continuous variables with a skewed distribution. Continuous variables were compared using the *t* test for independent variables. The Mann-Whitney test or the Kruskal-Wallis test was used for variables with a nonnormal distribution or for a small group size. The association between qualitative variables was assessed using the χ [2] test or Fisher’s exact test when a group was very small. Association of frailty with other geriatric syndromes and comorbidity was evaluated using the Cochran-Mantel-Haenszel test, and it was graphically represented by Venn diagram. We compared the estimated risk of all-cause mortality by VACS Index and the QOL of frail patients with the following four groups: those who have three or more comorbidities without frailty or other geriatric syndromes; those with one or more geriatric syndrome only; patients with geriatric syndromes and comorbidity without frailty; and patients with neither frailty, comorbidity, nor geriatric syndromes. For that purpose, comparing three or more groups simultaneously, the Bonferroni adjustment was applied to correct for a possible increase in type 1 errors (false positives). Statistical analysis was performed with SPSS Statistics for Windows (Version 25.0). All statistical tests were two-sided, and *P* values < 0.05 were considered statistically significant. The *P* set by Bonferroni was 0.05/5 because we made five comparisons, so *P* < .01 was considered statistically significant in this analysis. There was no imputation in the missing values; we worked with observed data.

## Results

Seven hundred and ninety-six patients were included, of whom 191 (24.1%) were female. The median age was 56.6 (53.7–61.1) years. Median years with known HIV infection was 21.5 (13.6–27.6), and 90.3% of cases were virologically controlled. Sociodemographic characteristics, HIV-infection-related factors, and data related to medications in this population have been previously published [4].

Frailty and prefrailty prevalence was 6 and 52.7%, respectively. The mean number of comorbidities was 2.2 (1.7) in the following order of frequency: dyslipidemia (43.8%), hypertension (28.7%), osteoarthritis (21.2%), diabetes mellitus (13.5%), COPD (10.9%), psychiatric disorders (9.2%), history of cancer (7.4%), cancer (6.9%), and chronic kidney disease (6.3%). More than one-third (38.3%) of patients had three or more of the listed comorbidities, and 389 (49%) patients had at least one of the following geriatric syndromes: falls, depression, cognitive impairment, and risk of malnutrition. The prevalence of each syndrome was 15.6, 26.8, 12.1 and 18.1%, respectively. QOL was reported to be fair or poor in 57.7% patients, the median VACS Index score was 22 (17–29), and the median estimated risk of all-cause 5-year mortality according to the VACS Index was 7.8% (5.8–11.3). Differences by sex are shown in Table 1. It is worth noting that women had significantly more comorbidity and geriatric syndromes and their estimated risk of all-cause 5-year mortality by VACS Index was significantly higher than in men.

**Table 1:** Frailty, comorbidity, geriatric syndromes, mortality, and QOL in older adults with HIV by sex

	Total	Men	Women	*p*
Patients. *N* (%)	796	602 (75.9)	194 (24.1)	
Frailty				
Frailty. *N* (%)	46 (5.8)	35 (5.8)	11 (5.7)	1
Prefrailty. *N* (%)	417 (52.7)	311 (51.6)	106 (54.6)	0.50
Comorbidity				
≥ 3 comorbidities. *N* (%)	306 (38.4)	218 (36.2)	88 (45.4)	0.02
Hypertension. *N* (%)	228 (28.7)	182 (30.2)	46 (23.8)	0.09
Type 2 Diabetes. *N* (%)	107 (13.5)	89 (14.9)	18 (9.3)	0.04
Dyslipidemia. *N* (%)	348 (43.8)	259 (43.2)	89 (45.9)	0.50
Osteoarthritis. *N* (%)	167 (21.2)	102 (17.1)	65 (34)	0.01
Chronic renal failure. *N* (%)	50 (6.3)	40 (6.7)	10 (5.2)	0.60
Cancer. *N* (%)	55 (6.9)	43 (7.2)	12 (6.2)	0.70
History of cancer. *N* (%)	59 (7.4)	34 (5.7)	25 (12.9)	0.01
COPD. *N* (%)	87 (11)	63 (10.5)	24 (12.5)	0.40
Psychiatric disorders. *N* (%)	73 (9.3)	51 (8.6)	22 (11.4)	0.20
Geriatric Syndromes				
≥ 1 geriatric syndrome. *N* (%)	389 (48.9)	272 (45.2)	117 (60.3)	0.01
Falls. *N* (%)	124 (15.6)	81 (13.5)	43 (22.2)	0.01
Depression. *N* (%)	213 (26.8)	153 (25.5)	60 (30.9)	0.10
Cognitive impairment. *N* (%)	96 (12.1)	71 (11.8)	25 (12.9)	0.70
Malnutrition risk. *N* (%)	143 (18.1)	101 (16.9)	42 (21.8)	0.10
Quality of life				
Fair or poor. *N* (%)	(57.7)	337 (55.7)	124 (63.3)	0.06
Mortality				
VACS Index score. Median (p25-p75)	22 (17–29)	18 (12–29)	23 (18–33)	0.01
Estimated risk of all-cause 5-year mortality by VACS Index. Median (p25-p75)	7.8 (5.8–11.3)	6.2 (4.2–11.3)	8.4 (6.2–13.8)	0.01

Frailty and prefrailty defined according to Frailty Phenotype. The GS considered were falls, cognitive impairment, depression, and risk of malnutrition. Cognitive impairment defined as MOCA test score < 20 points. Depression defined as SF-GDS score ≥ 6 points. Falls: at least one fall in the last year. Risk of malnutrition defined as MNA-SF score < 11 points. QOL evaluated by self-assessment and categorized into very good, good, fair, or poor. The Veterans Aging Cohort Study Index is a score created by summing pre-assigned points for age, CD4 count, HIV-1 RNA, hemoglobin, platelets, AST, ALT, creatinine, and viral hepatitis C infection. The higher the score the higher the risk of all-cause mortality. The risk can be estimated using the VACS index calculator (https://www.mdcalc.com/calc/2201/veterans-aging-cohort-study-vacs-1.0-index)

### Prevalence of frailty, geriatric syndromes, and comorbidity

**Fig. 1:**
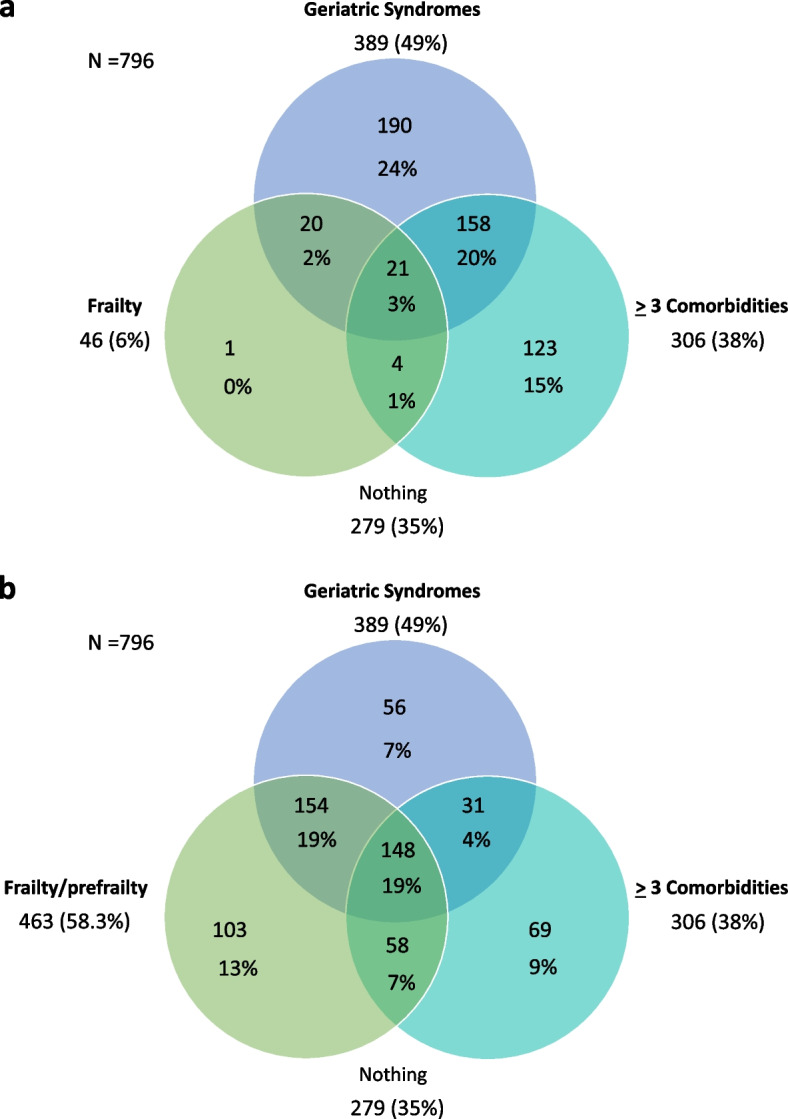
Prevalence and overlap of frailty, geriatric syndromes, and comorbidity in older adults with HIV. **a** Frailty vs non-frailty. **b** Frailty/prefrailty vs robust. Frailty and prefrailty defined according to Frailty Phenotype. Geriatric Syndromes were considered when the patient had at least one of the following: falls, cognitive impairment, depression, and risk of malnutrition. Falls considered whether the patient had at least one fall in the last year. Cognitive impairment defined as MOCA test score < 20 points. Depression defined as SF-GDS score ≥ 6 points. Risk of malnutrition defined as MNA-SF score < 11 points. Comorbidities were recorded due to self-reported, physician-diagnosed chronic conditions: hypertension, type 2 diabetes, dyslipidemia, coronary heart disease, stroke, COPD, chronic kidney disease, cancer (< 5 years from the diagnosis), history of cancer (≥ 5 years from the diagnosis; not active disease), psychiatric disorders, and osteoarticular disease

The prevalence and overlap of frailty, geriatric syndromes, and comorbidity is shown in Fig. 1. Frailty has been considered in two ways: frailty versus nonfrailty (Fig. 1a) and frailty/prefrailty versus robust (Fig. 1b). From the total, considering frailty versus nonfrailty, 279 (35%) patients had neither comorbidities, geriatric syndromes, nor frailty, but 517 (65%) did, and 211 (26.5%) patients had frailty and/or geriatric syndromes without any comorbidity.

### Effect of frailty, comorbidity, and geriatric syndromes on mortality

**Fig. 2:**
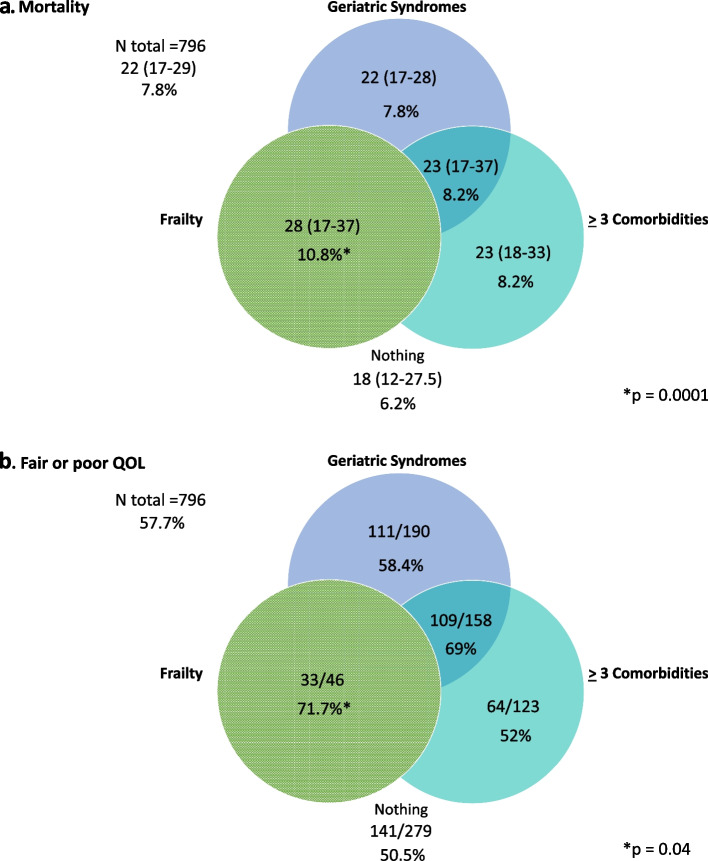
Risk of all-cause 5-year mortality and quality of life of frail patients compared with non-frail. **a** Mortality. **b** Fair or poor QOL. Non-frail patients were divided in four groups: patients with only > 1 geriatric syndrome (light grey); patients with only > 3 comorbidities (medium grey); patients with geriatric syndromes and comorbidity (dark grey), and those without frailty, comorbidity, and geriatric syndromes (out of the Venn’s Diagram). The risk of all-cause 5-year mortality was calculated with Veterans Aging Cohort Study Index (VACS Index) and expressed as percentage of probability. The median VACS Index score was expressed as absolute number (p25-p75). QOL is expressed by the number of OAWH reporting fair or poor QOL and the proportion it represents in each group

Results are shown in Table 2. Frailty, having more than three comorbidities, or having one or more geriatric syndromes was statistically and separately associated with higher risk of all-cause 5-year mortality calculated by VACS Index. Adding frailty to geriatric syndromes and/or comorbidity significantly increases the VACS score and the prediction of mortality. We compared the risk of all-cause 5-year mortality of frail patients with that of nonfrail patients (see Fig. 2a) whom we divided into four groups: patients with at least one geriatric syndrome; patients with only ≥ 3 comorbidities; patients with geriatric syndromes and comorbidity; and patients without frailty, comorbidity, and geriatric syndromes. The VACS Index score and the prediction of mortality for frail patients were significantly higher than they were for patients with geriatric syndromes and/or comorbidity.

**Table 2:** Mortality and QOL by frailty, comorbidity and/or geriatric syndromes in older adults with HIV

	Estimated risk of all-cause 5-year mortality* by VACS index				Quality of life		
	N	VACS index. Median (p25-p75)	%*	p	Good or very good	Fair or poor	p
					N (%)	N (%)	
Frailty							
Non frail	746	19 (17–29)	6.5	0.01	327 (43.5)	425 (56.5)	0.04
Frail	45	28 (18–37)	10.8		13 (28.3)	33 (71.7)	
Comorbidities							
< 3	485	18 (12–28)	6.2	0.01	223 (45.5)	267 (54.5)	0.02
≥ 3	306	23 (18–33)	8.2		115 (37.3)	193 (62.7)	
Geriatric Syndromes							
None	406	18 (12–29)	6.2	0.01	201 (49)	209 (51)	0.01
At least 1	387	23 (17–30)	8.2		139 (35.5)	252 (64.5)	
Frailty + GS							
No	753	20 (17–29)	6.9	0.01	330 (43.4)	430 (56.6))	0.02
Yes	40	28.5 (17.5–37.5)	11.1		10 (24.4)	31 (75.6)	
Frailty + Comorbidity							
No	768	22 (17–29)	7.8	0.01	333 (42.9)	443 (57.1)	0.1
Yes	25	29 (22–39)	11.3		7 (28)	18 (72)	
GS + Comorbidity							
No	615	18 (12–29)	6.2	0.001	286 (46.1)	335 (53.9)	0.01
Yes	178	23 (17–33)	8.2		54 (30)	126 (70)	
Frailty+ Comorbidity + GS							
No	772	22 (17–29)	7.8	0.01	335 (42.9)	445 (57.1)	0.1
Yes	21	30 (24–43)	11.9		5 (23.8)	16 (76.2)	
Frailty or GS							
No	401	18 (12–29)	6.2	0.01	198 (48.9)	207 (51.1)	0.02
Yes	392	23 (17–30)	8.2		142 (35.9)	254 (64.1)	
Cognitive impairment							
No	696	18 (17–29)	6.2	0.01	307 (43.7)	395 (56.3)	0.09
Yes	95	24 (17–33)	8.7		33 (34.4)	63 (65.6)	
Depression							
No	579	18 (14–29)	6.2	0.1	289 (49.5)	295 (50.5)	0.01
Yes	211	23 (17–29)	8.2		50 (23.5)	163 (76.5)	
Falls							
No	670	21 (17–29)	7.4	0.1	295 (43.8)	379 (56.2)	0.10
Yes	122	23 (17–30)	8.2		45 (36)	80 (64)	
Risk of malnutrition							
No	642	18 (17–29)	6.2	0.05	284 (43.8)	365 (56.2)	0.20
Yes	144	23 (17–33)	8.2		55 (38.2)	89 (61.8)	

QOL evaluated by self-assessment and categorized into very good, good, fair, or poor. Frailty defined according to Frailty Phenotype. VACS index: The Veterans Aging Cohort Study Index is a score created by summing pre-assigned points for age, CD4 count, HIV-1 RNA, hemoglobin, platelets, AST, ALT, creatinine, and viral hepatitis C infection. The higher the score the higher the risk of all-cause mortality. The risk can be estimated using the VACS index calculator https://vacs.med.yale.edu/calculator/IC. GS: Geriatric Syndromes. The GS considered were falls, cognitive impairment, depression, and risk of malnutrition. Cognitive impairment defined as MOCA test score < 20 points. Depression defined as SF-GDS score ≥ 6 points. Falls: at least one fall in the last year. Risk of malnutrition defined as MNA-SF score < 11 points

Among the four geriatric syndromes evaluated, the only one associated with higher risk of all-cause 5-year mortality was cognitive impairment (24 [17–33] vs. 18 [17–29], *P* = 0.01).

### Effect of frailty, comorbidity, and geriatric syndromes on QOL

Frailty, having three or more comorbidities, or one or more geriatric syndrome was statistically and separately associated with fair or poor QOL among OAWH. Results are shown in Table 2. We compared the QOL of frail patients with those nonfrail patients (see Fig. 2b) divided into four groups: patients with at least one geriatric syndrome; patients with ≥3 comorbidities; patients with geriatric syndromes and comorbidity; and patients without frailty, comorbidity, and geriatric syndromes. The QOL for frail patients was significantly worse than for patients with geriatric syndromes and/or comorbidity. The only geriatric syndrome that was significantly associated with worse QOL was depression (76.5% vs. 50.5%, *P* = 0.01).

## Discussion

Our study provides relevant data on the effect of frailty, comorbidity, and geriatric syndromes and their overlap on mortality and QOL in OAWH. We found they were separately associated with higher risk of all-cause 5-year mortality but the predicted risk of mortality for frail patients was significantly higher than it was for patients with geriatric syndromes and/or comorbidity. Furthermore, frailty combined with geriatric syndromes and/or comorbidity significantly increases the risk of all-cause 5-year mortality. Frailty has been previously associated with significantly higher all-cause mortality among OAWH [11, 31], and frailty observed in two consecutive visits in the follow-up was associated with an almost six-fold increased risk of death compared with those who maintained a robust state among aging persons with HIV and injection drug use [12]. In the older population overall, the combination of functional impairment and geriatric syndromes predicts health outcomes, including mortality, better than chronic comorbidities do, which implies that accounting for chronic conditions alone is not enough in the approach to treating older adults [32]. However, the literature still has no evidence showing the relationship between geriatric syndromes and mortality among OAWH. Our study provides information in this regard because we found that the risk of mortality from those with at least one geriatric syndrome was significantly higher than it was for those without them, and no differences were found with the patients with only comorbidity. The possible mechanism for explaining why frailty and geriatric syndromes are associated with the risk of mortality is that they are the expression of decreased reserves and high vulnerability to stressors because of a cumulative decline in key physiological and biological systems, such as the stress-response, metabolic, and musculoskeletal systems, that are crucial for maintaining homeostasis and, ultimately, life [33, 34]. Regarding comorbidity, the relationship between aging and disease is bidirectional. Aging is the major risk factor for most chronic diseases, and age-related diseases are the main pathway of pathological aging, which may accelerate biological aging and, in the end, death [35].

Regarding effects on QOL, frailty, comorbidity, and geriatric syndromes were all separately associated with fair or poor QOL, and the QOL for frail patients was significantly worse as well.

Frailty, comorbidity, and geriatric syndromes are predictors not only of survival but specifically of disability-free years of life; all of them have deleterious effects on physical function [8, 24, 33]. Maintaining functional ability that allows the person to be independent is the core of satisfactory aging, and, on the contrary, physical impairment is strongly associated with poor quality of life [7]. A recent systematic review and meta-analysis demonstrated that QOL is substantially worse for community-dwelling older people living with frailty [36]. Enhancing QOL should be a priority, especially for older adults and particularly for OAWH. In 2016, it was suggested that QOL be added as the “fourth 90” [37] to the UNAIDS “90–90-90” strategy [38] to ensure that the objective goes beyond viral suppression, but achieving this goal is far off. QOL is not routinely assessed in clinical practice, and the scientific evidence regarding the possible effect of frailty and geriatric syndromes on QOL is practically symbolic [22].

One in every four of the OAWH in our study had frailty and/or geriatric syndromes without significant comorbidity, and 49% of the total had at least one geriatric syndrome. This is interesting because comorbidity is routinely assessed and mostly well addressed in HIV clinics, but frailty and geriatric syndromes are not. Guidelines of management and treatment of patients with HIV are clear about the screening, approach, and treatment of the most prevalent comorbidities [39, 40], but frailty and geriatric syndromes are not mentioned except in a few cases [41, 42]. Due to their effect on mortality and QOL, at least their screening should be included in the clinical approach of OAWH. Frailty is potentially reversible [34], and the four geriatric syndromes evaluated (cognitive impairment, depression, falls, and risk of malnutrition) should be addressed and treated if detected. Interestingly, in the general population, geriatric syndrome information has been demonstrated to be especially helpful to understanding survival for younger old persons, more than in the very old [43]. Considering the premature appearance of frailty and geriatric syndromes in OAWH [44, 45], age 50 may be an appropriate age to begin screening, as recommended by the European AIDS Clinical Society Guidelines [41]. Cognitive impairment was associated in our cohort with higher risk of all-cause 5-year mortality. This association has been largely demonstrated in the overall population, leading to a 2.6-fold increase in the risk of death in dementia patients [46]. Regarding people with HIV in the post-antiretroviral treatment era, OAWH have a higher prevalence of risk factors for dementia than the overall population has in relation to both traditional and HIV-specific risk factors [47]. Recent works have published the association of cognitive impairment with mortality [48, 49], and a high burden of other geriatric syndromes has been demonstrated in OAWH with symptomatic cognitive impairment [50]. Depression is a well-known factor associated with poor QOL in people with HIV, and this relationship was found in our work as well. Consequently, treating OAWH should involve screening for at least these two geriatric syndromes (cognitive impairment and depression), in addition to frailty.

It is worth highlighting our finding that older women with HIV suffer a greater burden of comorbidity and geriatric syndromes than men do, with special mention of osteoarthritis and falls, two conditions that are risk factors for disability. No data with which to contrast our results are available. Osteoarthritis is a highly prevalent and disabling condition in older adults but is not usually recorded nor screened for among OAWH in research. In 2020, a scoping review of falls in people with HIV was published, but without any reference to possible differences in the falls’ prevalence by sex [51]. The comorbidity profile is clearly different between men and women, with a higher burden of cardiovascular risk factors among men and a higher rate of cancer history among women [5].

Our study has limitations that should be considered. Because it is inherent to observational research, a relationship of causality between variables cannot be established, and we evaluated risk of mortality instead of the evidence of death because it was a cross-sectional study. However, our study has great strengths, such as the number of OAWH included, the extensive assessment performed on all of them, and that it combined frailty, geriatric syndromes, and comorbidity to compare their effect on mortality and QOL, concluding that frailty had the greatest negative effect out of the three factors.

## Conclusions

Frailty, geriatric syndromes, and comorbidity had negative effects on mortality and QOL, but frailty had the greatest negative effect out of the three factors. This study is translational, and its results should be a wake-up call to standardize the screening for frailty and geriatric syndromes in OAWH in the clinical practice.

## Data Availability

The datasets used and/or analysed during the current study are available from the corresponding author on reasonable request.
